# P089 Jaw claudication without headache: a missed presentation of giant cell arteritis resulting in bilateral blindness

**DOI:** 10.1093/rap/rkag113.119

**Published:** 2026-11-03

**Authors:** Sadique Khan, Salema Khalid

**Affiliations:** Basingstoke and North Hampshire Hospital, Basingstoke, United Kingdom; Basingstoke and North Hampshire Hospital, Basingstoke, United Kingdom

## Abstract

**Introduction:**

Giant cell arteritis (GCA) is a granulomatous vasculitis affecting large- and medium-sized arteries, with a predilection for branches of the common carotid artery. Irreversible visual impairment occurs in 8%–19% of patients, with bilateral blindness reported in 2.7%–5.1%. Classical cranial symptoms, particularly headache, may be absent, delaying diagnosis and treatment. Although jaw claudication is a recognised predictor of visual loss, it may not immediately raise suspicion for GCA when headache is absent. We describe a patient in whom jaw claudication without headache was not initially recognised as GCA, with the diagnosis established only after rapidly progressive bilateral visual loss.

**Case description:**

An 82-year-old man with type 2 diabetes mellitus, hypertension, hypercholesterolaemia and ischaemic heart disease initially presented to his GP with bilateral jaw pain on chewing, restricted mouth opening and reduced oral intake resulting in weight loss. His GP suspected possible head and neck malignancy and referred him urgently to ENT.

A few days later, he developed progressive bilateral visual deterioration and re-presented to his GP, who referred him to an optician. His visual symptoms were attributed to bilateral cataracts and pre-existing retinal pathology.

He subsequently attended the Emergency Department with sudden-onset “fog-like” visual disturbance, worse in the right eye. No inflammatory markers or neuroimaging were performed, and he was discharged with ophthalmology review the following day.

The next morning, he awoke with complete bilateral visual loss, with no perception of light in either eye. Ophthalmological assessment demonstrated right anterior ischaemic optic neuropathy and left central retinal artery occlusion with a faint cherry-red spot and retinal thinning, highly suggestive of GCA. He was commenced on intravenous methylprednisolone 1 g daily for three days and referred to rheumatology.

Investigations demonstrated CRP 157, Hb 127, platelets 435, but ESR of 5. He had no headache, scalp tenderness or symptoms of polymyalgia rheumatica. Examination revealed bilaterally thickened temporal arteries with preserved pulsation, although the left pulse was reduced. CT head, CT angiography of the head and neck, and CT chest, abdomen and pelvis excluded acute cerebrovascular pathology and underlying malignancy. Temporal artery ultrasound demonstrated bilateral halo signs with arterial wall thickening and coexistent arterial calcification. Temporal artery biopsy confirmed GCA.

Despite treatment with high dose steroids, his bilateral visual loss remained irreversible.

**Discussion:**

Our case illustrates the diagnostic challenge of GCA when classical cranial symptoms are absent. Headache, the commonest presenting feature, occurs in approximately two-thirds of patients, leaving around one-third who present without headache. Jaw claudication is reported in approximately half of patients. Constitutional symptoms, including fever, fatigue, anorexia and weight loss are also variable, making atypical presentations an important cause of delayed diagnosis.

Increasing age, male sex, hypertension, jaw claudication, delayed initiation of glucocorticoid therapy and a positive temporal artery biopsy have all been associated with an increased risk of visual loss. Furthermore, patients without polymyalgia rheumatica are approximately three times more likely to develop permanent visual loss, while those without headache are more than four times more likely than those presenting with headache. Our patient exhibited all of these high-risk features.

Despite biopsy-proven GCA, our patient had a normal ESR with markedly elevated CRP and platelet count. CRP has been shown to be more sensitive than ESR, and discordance between ESR, CRP and platelet count is well recognised. Evidence suggests that combining CRP and platelet count provides greater diagnostic utility than ESR alone, with ESR contributing little additional diagnostic value when interpreted alongside these markers.

Extensive vascular calcification on temporal artery ultrasound and significant cardiovascular comorbidity initially raised concern for alternative vascular pathology. Nevertheless, coexistent atherosclerosis should not lower suspicion for GCA when the clinical presentation is suggestive.

Lastly, visual prognosis following GCA-related visual loss remains poor despite prompt treatment, with meaningful visual recovery reported in only approximately 4% of affected patients. In another study, no patients developed visual loss when high-dose corticosteroids were started before ocular involvement. However, among patients presenting with established visual loss, approximately 10% experienced further visual deterioration despite treatment, usually within the first five days.

**Key learning points:**

• This case reinforces several important clinical learning points.

• First, clinicians should maintain a high index of suspicion for GCA in elderly patients presenting with jaw claudication or unexplained visual symptoms, even when classic symptoms like headache, scalp tenderness or polymyalgia rheumatica are absent.

• Second, this patient’s multiple healthcare encounters before diagnosis illustrate the need for improved recognition of atypical GCA presentations across specialties including primary care and emergency medicine.

• Third, inflammatory markers should be interpreted collectively rather than individually. A normal ESR should not be used to exclude GCA, particularly when CRP and platelet count are elevated and the clinical presentation is suggestive. Temporal artery ultrasound should be performed early where available and supported by temporal artery biopsy to confirm the diagnosis.

• Fourth, although significant cardiovascular disease and coexistent atherosclerosis may raise concern for alternative vascular pathology, investigation of these conditions should occur alongside assessment for GCA.

• Finally, because visual recovery after established GCA-related blindness is uncommon, the greatest opportunity to preserve vision lies in recognising high-risk clinical features and commencing high-dose glucocorticoid therapy before irreversible ischaemic injury has occurred.

